# Epinephrine Activation of the β_2_-Adrenoceptor Is Required for IL-13-Induced Mucin Production in Human Bronchial Epithelial Cells

**DOI:** 10.1371/journal.pone.0132559

**Published:** 2015-07-10

**Authors:** Nour Al-Sawalha, Indira Pokkunuri, Ozozoma Omoluabi, Hosu Kim, Vaidehi J. Thanawala, Adrian Hernandez, Richard A. Bond, Brian J. Knoll

**Affiliations:** 1 Department of Pharmacological and Pharmaceutical Sciences, University of Houston, 4800 Calhoun Road, Houston, Texas, 77204, United States of America; 2 Department of Biology and Biochemistry, University of Houston, 4800 Calhoun Road, Houston, Texas, 77204, United States of America; University of Alabama at Birmingham, UNITED STATES

## Abstract

Mucus hypersecretion by airway epithelium is a hallmark of inflammation in allergic asthma and results in airway narrowing and obstruction. Others have shown that administration a T_H_2 cytokine, IL-13 is sufficient to cause mucus hypersecretion *in vivo* and *in vitro*. Asthma therapy often utilizes β_2_-adrenoceptor (β_2_AR) agonists, which are effective acutely as bronchodilators, however chronic use may lead to a worsening of asthma symptoms. In this study, we asked whether β_2_AR signaling in normal human airway epithelial (NHBE) cells affected mucin production in response to IL-13. This cytokine markedly increased mucin production, but only in the presence of epinephrine. Mucin production was blocked by ICI-118,551, a preferential β_2_AR antagonist, but not by CGP-20712A, a preferential β_1_AR antagonist. Constitutive β_2_AR activity was not sufficient for IL-13 induced mucin production and β-agonist-induced signaling is required. A clinically important long-acting β-agonist, formoterol, was as effective as epinephrine in potentiating IL-13 induced MUC5AC transcription. IL-13 induced mucin production in the presence of epinephrine was significantly reduced by treatment with selective inhibitors of ERK1/2 (FR180204), p38 (SB203580) and JNK (SP600125). Replacement of epinephrine with forskolin + IBMX resulted in a marked increase in mucin production in NHBE cells in response to IL-13, and treatment with the inhibitory cAMP analogue Rp-cAMPS decreased mucin levels induced by epinephrine + IL-13. Our findings suggest that β_2_AR signaling is required for mucin production in response to IL-13, and that mitogen activated protein kinases and cAMP are necessary for this effect. These data lend support to the notion that β_2_AR-agonists may contribute to asthma exacerbations by increasing mucin production via activation of β_2_ARs on epithelial cells.

## Introduction

Asthma is a chronic inflammatory disease characterized by airway hyperreactivity, subepithelial fibrosis, airway smooth muscle hyperplasia and mucous metaplasia [1]. Mucous metaplasia is an increase in the number of mucus-secreting goblet cells in the epithelium [2] that results in increased mucus synthesis and secretion. Excessive accumulation of airway mucus leads to the formation of mucous plugs that reduce the effective airway diameter and increase airway resistance. Patients who die of severe asthma attacks often exhibit goblet cell hyperplasia, mucus accumulation and large mucus plugs of unusual solidity due to high mucin content in their peripheral airways compared to asthmatic patients who did not die of acute attacks [3].

Allergic asthma has properties of a type I hypersensitivity, in which type 2 T-helper lymphocytes and type 2 innate lymphoid cells contribute to produce a distinctive set of cytokines in the airways, including IL-5, IL-9 and IL-13 [4]. Although the allergic airway also contains diverse hematopoietic and parenchymal cells, and factors secreted by them, airway epithelial overexpression of IL-13 or airway instillation of IL-13 is sufficient to induce mucous metaplasia in mice [5, 6]. Airway epithelium is essential and sufficient for mucous metaplasia induced by IL-13, and this is dependent on the expression of STAT6 (which mediates the action of IL-13) in the epithelium [7]. The epithelium has been suggested by many studies to play an important role in the pathogenesis of asthma [7–10] as well as being a key contributor to the mucus plugs responsible for asthma mortality [11]. Sputum of asthmatic patients show elevated levels of IL-13 and its presence is negatively associated with therapeutic responsiveness [12].

The MUC5AC gene encodes the major component of mucin in human airways, and induction of MUC5AC transcription by IL-13 is observed in cultured human airway epithelium [10, 13]. However other factors also are required for MUC5AC transcription in these cells. Signaling through the EGF [10] and TGF-β2 [14] receptors is required for IL-13 to induce MUC5AC transcription, and in the promoter region of the gene there are binding sites for numerous diverse transcription factors [15], although none for STAT-6, suggesting the action of multiple intersecting and possibly indirect pathways. Recently, we found evidence for the involvement of β-adrenoceptor (β_2_AR) signaling in the pathogenesis of asthma. Pharmacologic or genetic ablation of β_2_AR signaling causes reductions in mucous metaplasia, airway cellularity and airway hyperreactivity (AHR) in a murine asthma model [16, 17]. Thus, among the other pathways already mentioned, some that are initiated or influenced by β_2_ARs may also be involved in the regulation of MUC5AC transcription.

Due to the complexity of the signaling pathways that are involved in mediating mucus production, and the involvement of diverse cell types in whole animal models, we undertook to study human airway epithelial cells cultured in low concentrations of retinoic acid, conditions where mucin expression is normally minimal. We investigated the requirement for β_2_AR signaling in the transcription of the MUC5AC gene, the expression of MUC5AC protein and intracellular mucin accumulation in response to IL-13. In addition, we examined signaling components downstream of β_2_AR that may be required for this response.

## Materials and Methods

### Cell Culture

Normal human bronchial epithelial (NHBE) cells were obtained from Lonza (Walkersville, MD). The cells were seeded on Transwell-culture inserts (0.45 μm pore size) at 2 x 10^4^ / cm^2^ and grown in 5% CO_2_/95% air at 37°C in differentiation medium: 50% bronchial epithelial basal media, 50% DMEM high glucose and supplemented with 30 μg/ml bovine pituitary extract, 0.5 μg/ml BSA, 0.5 μg/ml epinephrine, 50 μg/ml gentamycin, 50 ng/ml amphotericin B, 0.5 ng/ml human EGF, 0.5 μg/ml hydrocortisone, 5 μg/ml insulin, 7 ng/ml triiodothyronine, 10 μg/ml transferrin and 0.1 ng/ml retinoic acid The cells were cultured with epinephrine for ~8 days until they reached confluence, then the apical medium was removed and air-liquid interface (ALI) was established. The cells were then treated with 20 ng/ml of IL-13 combined with different antagonists or inhibitors. In some experiments, the cells were grown in the absence of epinephrine 72 hours before they reached ALI. Compound-related toxicity was assessed through the dryness of the apical surface of the cultured NHBE cells [18]. Cells grown in the absence or presence of epinephrine formed tight junctions that produced equal increases in transepithelial electrical resistance (TEER) suggesting similar epithelial barrier function, consistent with previously published findings [19]. The formation of tight junctions was also confirmed by the presence of ZO-1 on the cell peripheries by immunofluorescence (data not shown).

### Real-Time PCR Analysis of MUC5AC Expression

Total RNA was extracted from cells using Trizol according to manufacturer’s protocol. cDNA was generated from 5 μg of total RNA and MUC5AC and 18S mRNA were quantified using the Taqman Gene Expression Assay (Applied Biosystems, Grand Island, NY) and analyzed by real time quantitative PCR (ABI PRISM 7000 Sequence Detection System, Applied Biosystems). The threshold cycles (C_t_) for MUC5AC of treated groups was compared to the control groups and normalized to 18S. Relative MUC5AC expression was calculated using Delta-Delta CT method.

### Immunoblotting

NHBE cells were lysed in a buffer containing 20 mM Tris-HCl (pH 7.5), 150 mM NaCl, 1 mM Na_2_EDTA, 1 mM EGTA, 1% Triton, 2.5 mM sodium pyrophosphate, 1 mM β-glycerophosphate, 1 mM Na_3_VO_4_ and 1 μg/ml leupeptin (Cell Signaling, Danvers, MA) combined with protease inhibitor cocktail tablet (Roche Applied Sciences, Indianapolis, IN). Total protein concentration was determined by BCA Protein Assay Kit (Thermo Fisher Scientific Inc, Rockford, IL), according to the manufacture’s protocols. Thirty micrograms of protein per lane were subjected to SDS-PAGE using 10% Tris-HCl gels (Bio-Rad Laboratories, Hercules, CA) and transblotted to PVDF membrane (EMD Millipore, Billerica, MA). Membranes were blocked in 3% BSA for 1 hour at room temperature and then incubated with antibodies against phospho-ERK1/2 (Cell Signaling, #4370S), phospho-p38 (Santa Cruz Biotechnology, #sc-17852-R) or phospho-c-Jun (EMD Millipore, #06–659) overnight at 4°C followed by treatment for 1 hour with HRP conjugated secondary antibody. The protein bands were developed using SuperSignal West Pico chemiluminescent substrate (Thermo Fisher Scientific) according to manufacturer’s recommendations. A CCD camera (Fluorochem 8800) was used to collect the digital images and AlphaEase software (ProteinSimple, Santa Clara, CA) to quantify band density. The membranes were then stripped and probed with GAPDH antibody (EMD Millipore, #MAB374)). The signal density of the phosphorylated proteins was normalized with that of GAPDH.

### Periodic Acid Fluorescent Schiff’s (PAFS) Staining

The apical surfaces of NHBE cells were washed with PBS, fixed in 4% paraformaldehyde (PFA) and permeabilized with Triton X-100. The inserts were stained with PAFS as described previously [20]. Red fluorescence of mucin was detected when the slides were excited at 380–580 nm and observed at 600–650 nm while the nucleic acid and cytoplasm of the cells fluorescence green when observed at a lower wavelength (380–500 nm and 450–475 nm excitation and emission wavelengths respectively) [20]. Images were captured using an Olympus DUS spinning disc confocal microscope maintained in the College of Pharmacy Imaging Core. To correct for insert background, empty inserts were stained and used as a negative control. To maintain consistency in subsequent image analysis, we used the same channel-specific threshold when capturing all images. The ratio of integrated mucin density to integrated nucleic acid and cytoplasm density was calculated using Image J (NIH).

### Immunofluorescence Labeling for Mucin 5AC

The apical surfaces of NHBE cells were washed with PBS, fixed in 4% paraformaldehyde (PFA) and permeabilized with Triton X-100. The inserts were incubated for 15 minutes with 10% normal goat serum at room temperature followed by Mucin 5AC Antibody (H-160, Santa Cruz Biotechnology) [21] overnight at 4°C. After washing the inserts with PBS, they were incubated with Alexa 594 goat anti-rabbit secondary antibody for 1 hour at room temperature. DAPI, at final concentration of 1 μg/ml, was used to counterstain the nuclei. Slides incubated with primary antibody diluents were used as negative controls. Images were captured by confocal microscopy and the same channel-specific threshold was maintained when capturing the images. The ratio of integrated mucin 5AC density of each group to the integrated mucin 5AC density of the corresponding control group was calculated using Image J software (NIH).

### Statistical Analysis

Data are presented as means ± SEM. All experiments were done with NHBECs from 3 donors (N = 3). One-way ANOVA followed by Tukey's multicomparison test for multiple group statistical analysis was performed using GraphPad Prism 4 software. p<0.05 was considered statistically significant.

## Results

**Fig 1:**
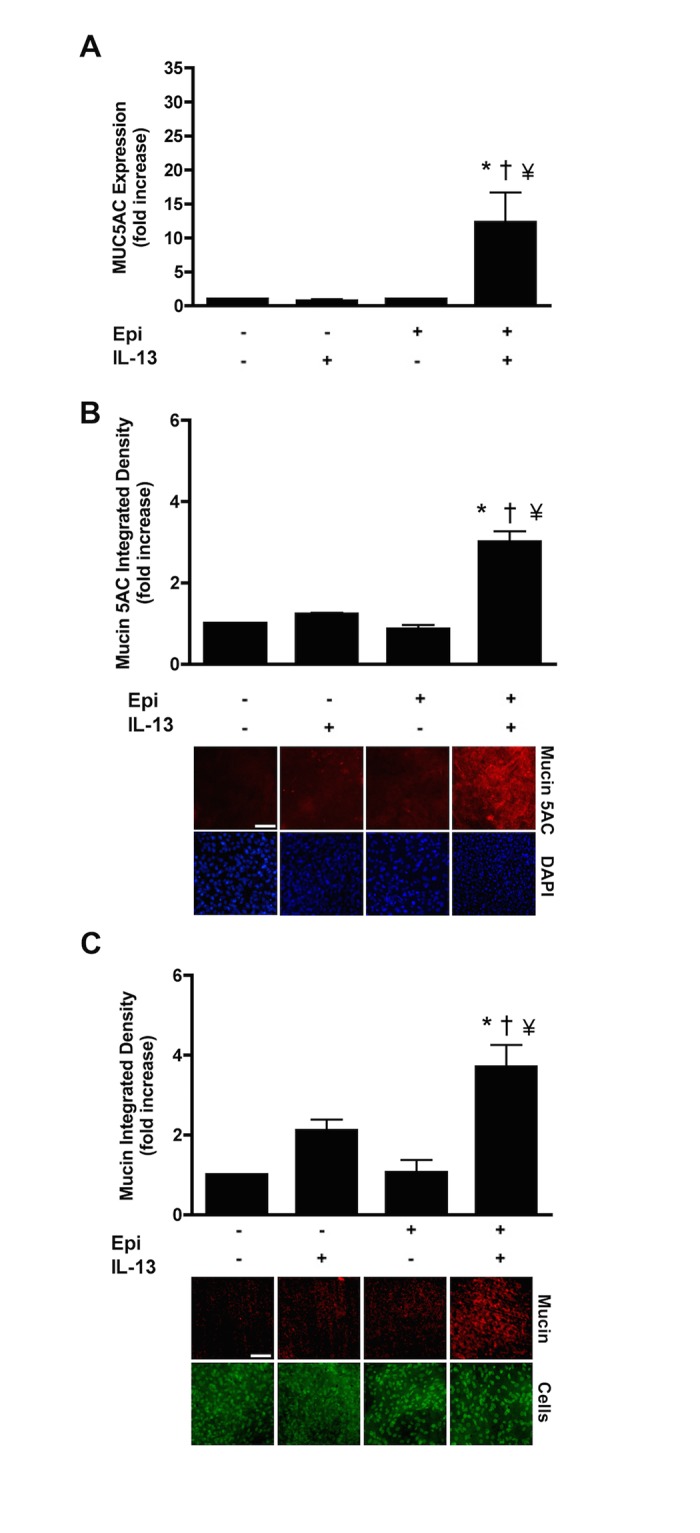
Epinephrine is required for mucin production in response to IL-13 in NHBE cells. **A**: NHBE cells were grown in the presence or absence of 3 μM epinephrine. At ALI, the cells were treated with 20 ng/ml IL-13 for 14 days, total RNA was harvested and then MUC5AC transcripts were measured by qRT-PCR. Data are presented as fold change compared to the corresponding treatment control (in the absence of IL13). **B**: Representative images of immunofluorescence with a rabbit antibody against human mucin 5AC (red) (scale bar = 100 μm). The Transwell membranes were incubated with DAPI to counterstain the nuclei (blue). Incubation with antibody diluent showed no red fluorescence (data not shown). The ratio of integrated fluorescence density of each group to the integrated mucin 5AC density of the corresponding control group was calculated and expressed as fold change. **C**: PAFS staining of NHBE cells to quantify total intracellular mucin glycoproteins. Representative images are shown. The ratio of mucin integrated density and nucleic acid/cytoplasm integrated density was calculated and the data presented as fold change compared to the corresponding control cells (in the absence of IL-13 treatment). Data are presented as means ± SEM from three donors. *, † and ¥ indicate p<0.05 significance as compared to + epinephrine,−epinephrine and −epinephrine + IL-13 treated cells respectively.

To determine whether epinephrine increases mucus production in NHBE cells, we cultured them for 72 hours before reaching ALI in a medium that lacks epinephrine and then stimulated with 20 ng/ml of IL-13 for 14 days, also in the absence of epinephrine. Under these conditions, IL-13 did not increase the expression of MUC5AC mRNA, and in the presence of epinephrine alone, MUC5AC mRNA was scarcely detectable above baseline. But with IL-13 treatment in the presence of epinephrine, the MUC5AC expression level was increased by ~15 fold (Fig 1A). Similar results were obtained with cells grown in the presence of 50 nM retinoic acid (S1 Fig). To correlate mRNA expression levels with intracellular mucin 5AC accumulation and mucin glycoprotein, immunofluorescence with anti-mucin 5AC antibody and PAFS staining were used respectively. Epinephrine was required for IL-13-induced increases in the intracellular mucin 5AC and total mucin glycoproteins (Fig 1B and 1C).

To determine if a therapeutic β-agonist showed an effect similar to epinephrine, we substituted formoterol. NHBE cells were cultivated with physiological levels of RA to more closely mimic the *in vivo* state. Under these conditions, IL-13 again did not significantly increase MUC5AC transcription (though levels were slightly higher). However the addition of formoterol increased MUC5Ac transcripts to a similar degree as epinephrine (S2 Fig).

**Fig 2:**
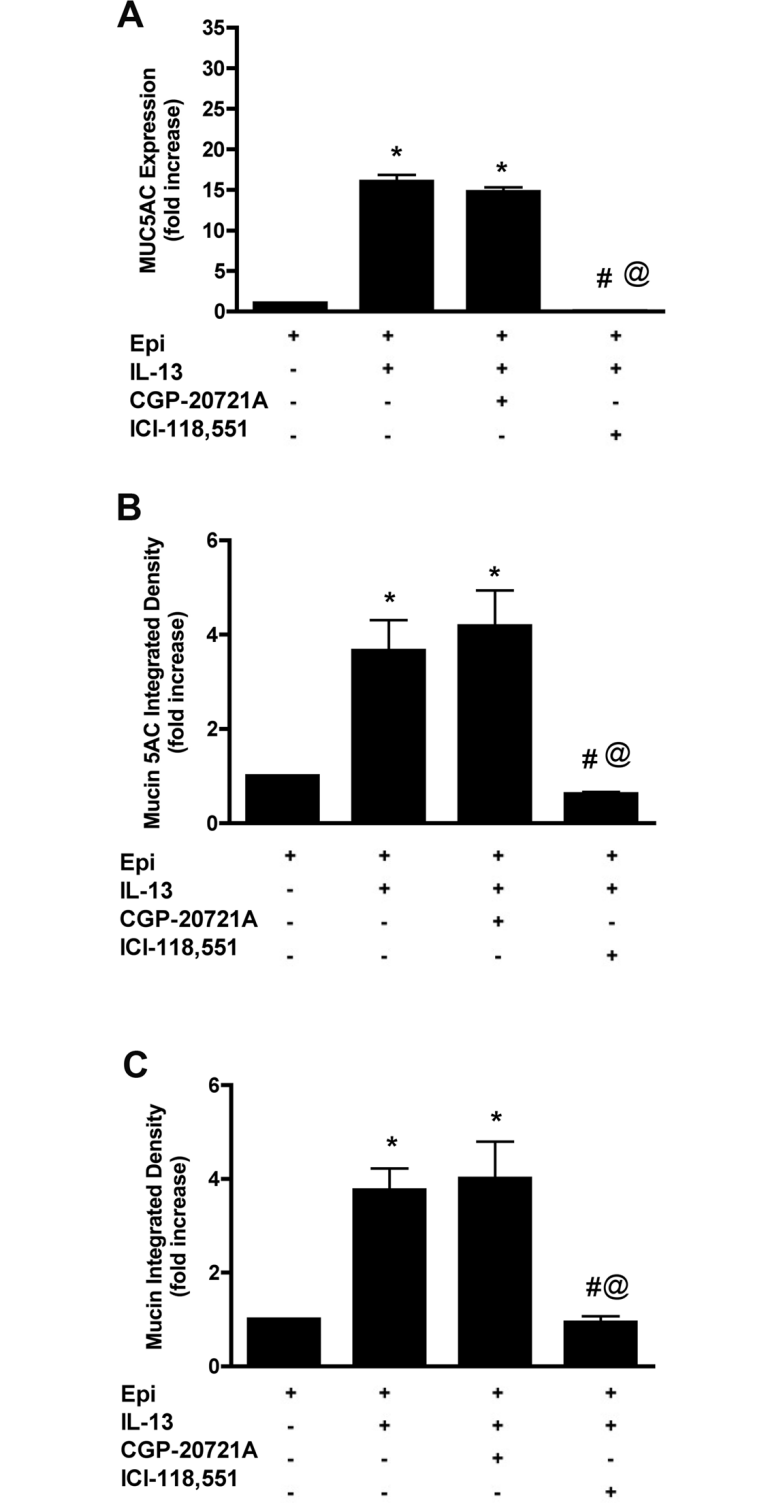
β_2_ARs are required for mucin production in response to IL-13 in NHBE cells. NHBE cells were grown in the presence of 3 μM epinephrine, then at ALI, they were treated with 20 ng/ml IL-13 in combination with 3 μM CGP-20712A (a preferential β_1_AR antagonist) or 1 μM ICI-118,551 (a preferential β_2_AR antagonist) for 14 days. **A**: MUC5AC transcripts were quantified from extracted total RNA by qRT-PCR. Data are presented as fold change compared to cells grown in the presence of epinephrine only. **B**: Quantification of intracellular mucin 5AC content. The ratio of mucin 5AC integrated density of each group to the integrated density of the cells grown in the presence of epinephrine alone (control cells) was calculated and expressed as fold change. See the supplement for the representative images (S3A Fig). **C**: Quantification of intracellular mucin glycoproteins in response to different ligands. The ratio of mucin integrated density and nucleic acid/cytoplasm integrated density was calculated and the data presented as fold change compared to control cells. See the supplement for the representative images (S3B Fig). Data are presented as means ± SEM from three donors. *, # and @ indicate p<0.05 significance as compared to + epinephrine, + epinephrine + IL-13 and + epinephrine + IL-13 + CGP-20721A treated cells respectively.

To determine the βAR subtype involved in MUC5AC expression in response to IL-13 in the presence of epinephrine, NHBE cells were incubated with either a preferential β_2_AR antagonist (1 μM ICI-118,551) or a preferential β_1_AR antagonist (3 μM CGP-20712A). ICI-118,551 completely abolished (>99%) IL-13 induced MUC5AC expression (0.039 ± 0.038 fold *vs* 15.99 ± 1.48 fold increase by IL-13. p<0.05). On the other hand, CGP-20712A did not affect the MUC5AC expression level (14.75 ± 0.96 fold *vs* 15.99 ± 1.48 fold increase by IL-13, p>0.05) (Fig 2A). CGP-20712A did not affect the intracellular mucin levels induced by IL-13 while ICI-118,551 brought the levels back to baseline (Fig 2B and 2C; for representative images see S3A and S3B Fig).

**Fig 3:**
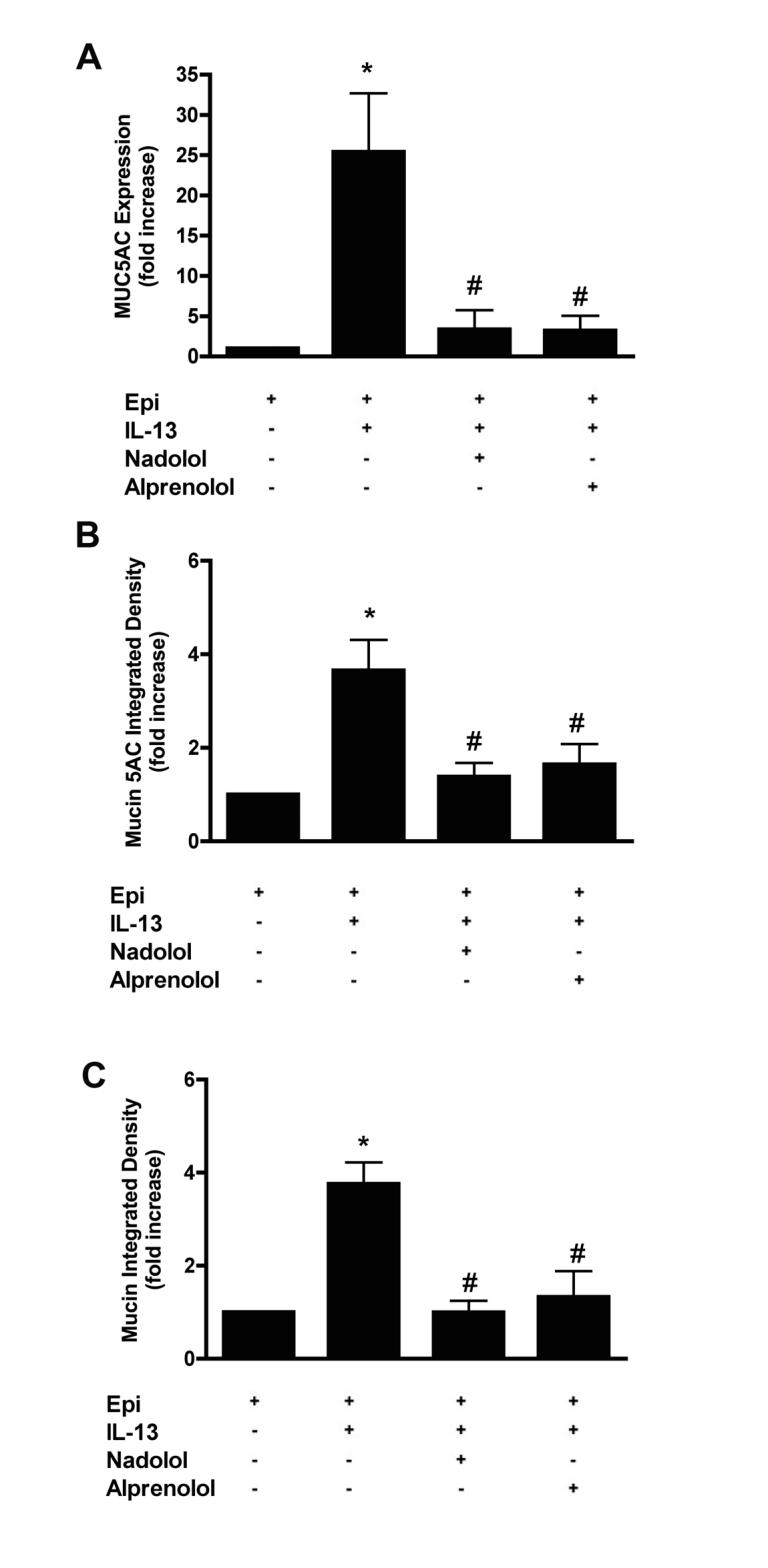
Agonist induced β_2_AR signaling is required for mucin production in response to IL-13 in NHBE cells. NHBE cells were grown in the presence of 3 μM epinephrine, then at ALI, they were treated with 20 ng/ml IL-13 in combination with 10 μM nadolol (a non-selective inverse agonist of βARs) or 10 μM alprenolol (a non-selective β_2_AR blocker with no inverse agonist activity) for 14 days. **A**: MUC5AC transcripts were measured by qRT-PCR. Data are presented as fold change compared to cells grown in the presence of epinephrine only. **B**: Quantification of intracellular mucin 5AC content. The ratio of mucin 5AC integrated density of each group to the integrated density of the cells grown in the presence of epinephrine alone (control cells) was calculated and expressed as fold change. See the supplement for the representative images (S4A Fig). **C**: Quantification of intracellular mucin glycoproteins in response to different ligands. The ratio of mucin integrated density and nucleic acid/cytoplasm integrated density was calculated and the data presented as fold change compared to control cells. See the supplement for the representative images (S4B Fig). Data are presented as means ± SEM from three donors. * and # indicate p<0.05 significance as compared to + epinephrine and + epinephrine + IL-13 treated cells respectively.

We next asked if the increased MUC5AC expression in response to IL-13 is due to agonist induced or constitutive β_2_AR signaling. NHBE cells were treated with 10 μM nadolol, a non-selective βAR ligand with inverse agonist activity at β_2_ARs that blocks both constitutive and agonist-induced receptor activity, or with 10 μM alprenolol, a non-selective βAR antagonist with no inverse agonist activity, for 14 days in combination with IL-13 and in the presence of epinephrine. Treatment with nadolol reduced IL-13 induced MUC5AC expression (3.36 ± 4.10 fold *vs* 25.37 ± 16.30 fold increase by IL-13, p<0.05), intracellular mucin 5AC protein and mucin content (Fig 3A, 3B and 3C; for representative images see S4A and S4B Fig). Treatment with alprenolol reduced IL-13-induced MUC5AC expression to a similar extent (3.19 ± 3.73 fold *vs* 25.37 ± 16.30 fold increase by IL-13, p<0.05) and also reduced intracellular mucin 5AC and mucin content (Fig 3A, 3B and 3C, and S4A and S4B Fig for representative images).

**Fig 4:**
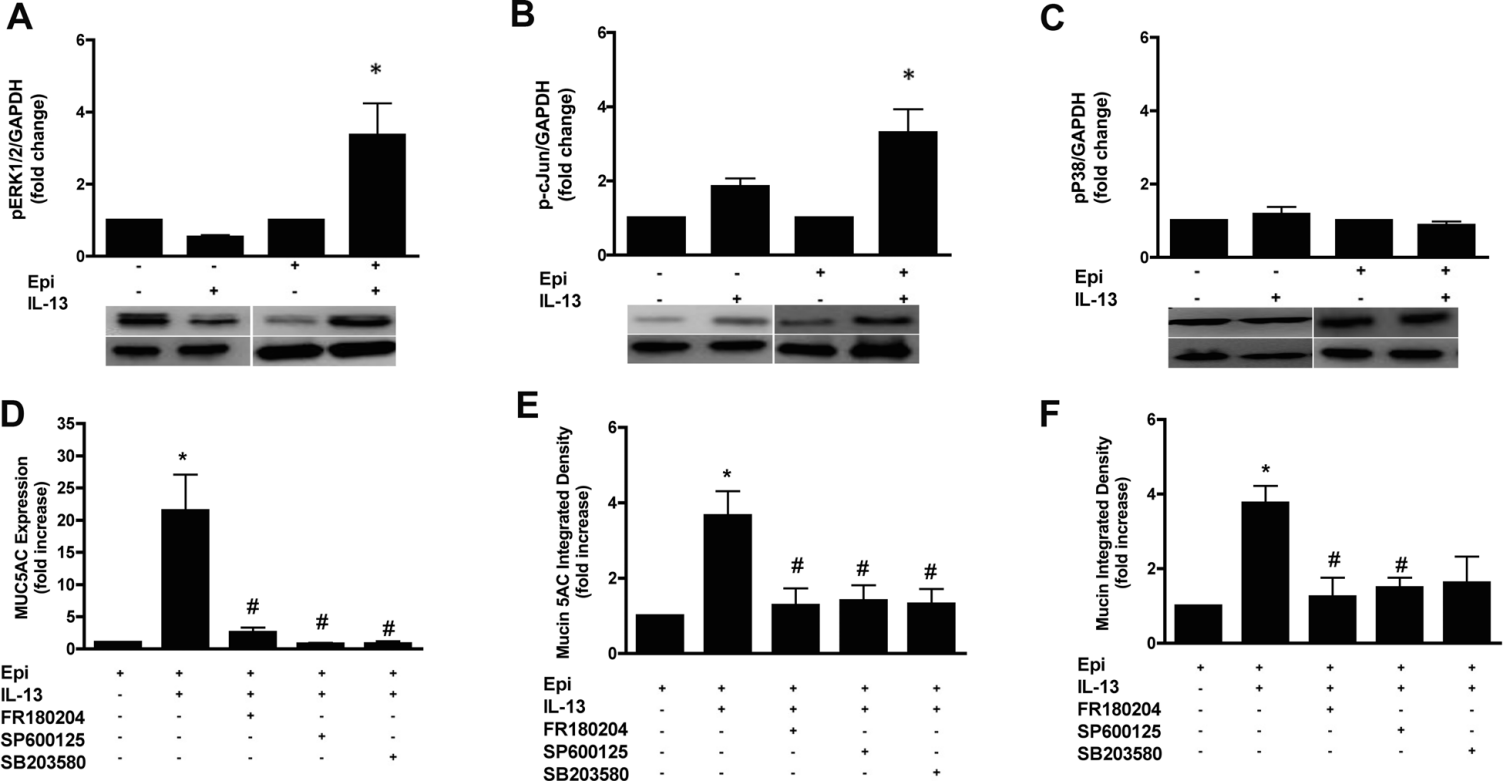
MAPK signaling is required for mucin production in response to IL-13 in NHBE cells. **A-C**. NHBE cells were grown in the presence or absence of epinephrine or IL-13 as indicated for 14 days after ALI before harvesting for total proteins. Immunoblots were performed using antibodies to the indicated phosphorylated MAP-kinases. The signal densities of the phosphorylated proteins were normalized to GAPDH protein density and the relative intensities were reported as the degree of activation of the protein. The data presented as fold change compared to the corresponding control cells. **D-F**: NHBE cells were grown in the presence of 3 μM epinephrine, then at ALI, they were treated with 20 ng/ml IL-13 in combination with 3 μM FR180204 (ERK1/2 inhibitor), 3 μM SP600125 (JNK inhibitor) or 3 μM SB203580 (p38 inhibitor) for 14 days. **D**: MUC5AC transcripts were measured by qRT-PCR, and the data presented as fold change compared to cells grown in the presence of epinephrine only. **E**: Quantification of intracellular mucin 5AC content. The ratio of mucin 5AC integrated density of each group to the integrated density of the cells grown in the presence of epinephrine alone (control cells) was calculated and expressed as fold change. See the supplement for the representative images (S5A Fig). **F**: Quantification of total intracellular mucin glycoproteins. The ratio of mucin integrated density and nucleic acid/cytoplasm integrated density was calculated and the data presented as fold change compared to control cells. See the supplement for the representative images (S5B Fig) Data are presented as means ± SEM from three donors. * and # indicate p<0.05 significance as compared to +epinephrine and + epinephrine + IL-13 treated cells respectively.

To investigate the role of mitogen activated protein kinases (MAPKs), we examined their activation using antibodies specific for phosphorylated (activated) MAPKs. In the absence of epinephrine, IL-13 did not affect the phosphorylation of ERK1/2 (Fig 4A), c-Jun (Fig 4B) or p38 (Fig 4C) as compared to their corresponding controls. When epinephrine was included in the medium, IL-13 induced an approximately 3-fold increase in the phosphorylation of ERK1/2 and c-Jun when compared to their corresponding controls (Fig 4A and 4B). However, phosphorylation of p38 was unaffected by IL-13 even in the presence of epinephrine (Fig 4C). Next, we treated NHBE cells with 3 μM FR180204, SP600125 or SB203580 (inhibitors of ERK1/2, JNK and p38 respectively) in combination with IL-13 and epinephrine for 14 days. All three MAPKs inhibitors significantly reduced MUC5AC gene expression (15.18 ± 3.76 fold increase by IL-13 vs 1.82 ± 0.68, 0.77 ± 0.39 and 0.80 ±0.65 fold by FR180204, SP600125 and SB203580 respectively) (Fig 4D). While all MAPK inhibitors reduced the intracellular mucin 5AC protein (see Fig 4E and S5A Fig for representative images), only FR180204 and SP600125 reduced intracellular mucin content when compared to IL-13 treated cells (see Fig 4F and S5B Fig for representative images).

**Fig 5:**
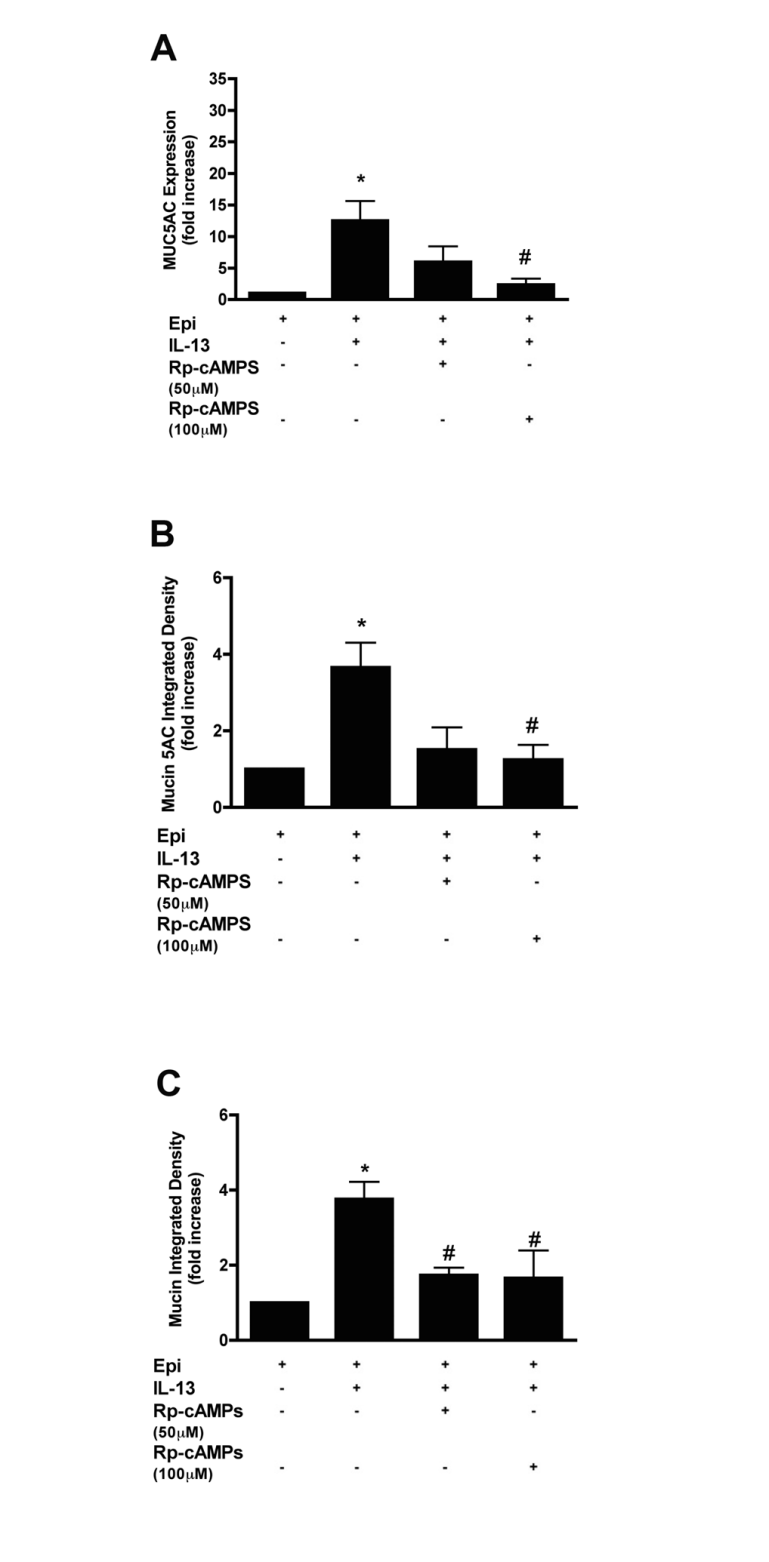
Inhibiting PKA signaling reduced mucin production in response to IL-13 in NHBE cells. Cells were grown in the presence of 3 μM epinephrine, then after ALI, they were treated with 20 ng/ml IL-13 in combination with 50 μM or 100 μM Rp-cAMP (cAMP-dependent protein kinase inhibitor) for 14 days. **A**: MUC5AC transcripts were measured by qRT-PCR, and the data presented as fold change compared to cells grown in the absence of inhibitor. **B**: Quantification of intracellular mucin 5AC content. The ratio of mucin 5AC integrated density of each group to the integrated density of the cells grown in the presence of epinephrine alone (control cells) was calculated and expressed as fold change. See the supplement for the representative images (S6A Fig). **C**: Quantification of intracellular mucin glycoproteins. The ratio of mucin integrated density and nucleic acid/cytoplasm integrated density was calculated and the data presented as fold change compared to control cells. See the supplement for the representative images (S6B Fig). Data are presented as means ± SEM from three donors. * and # indicate p<0.05 significance as compared to + epinephrine and + epinephrine + IL-13 treated cells respectively.

To explore a possible role for PKA in the induction of MUC5AC, we treated NHBE cells with a competitive cAMP analogue, Rp-cAMPS, for 14 days in combination with IL-13 and epinephrine. Rp-cAMPS did not significantly reduce the levels of MUC5AC expression at 50 μM (5.97 ± 4.29 fold *vs* 12.50 ± 5.38 fold increase by IL-13, p>0.05,) while at 100 μM, there was a significant reduction (2.35 ± 1.63 fold *vs* 12.50 ± 5.38 fold increase by IL-13, p<0.05)(Fig 5A). The intracellular mucin 5AC protein level was significantly reduced when the cells were treated with 100 μM Rp-cAMPS but not at 50 μM, while mucin glycoproteins levels were reduced at both concentrations (see Fig 5B and 5C, and S6A and S6B Fig for representative images).

**Fig 6:**
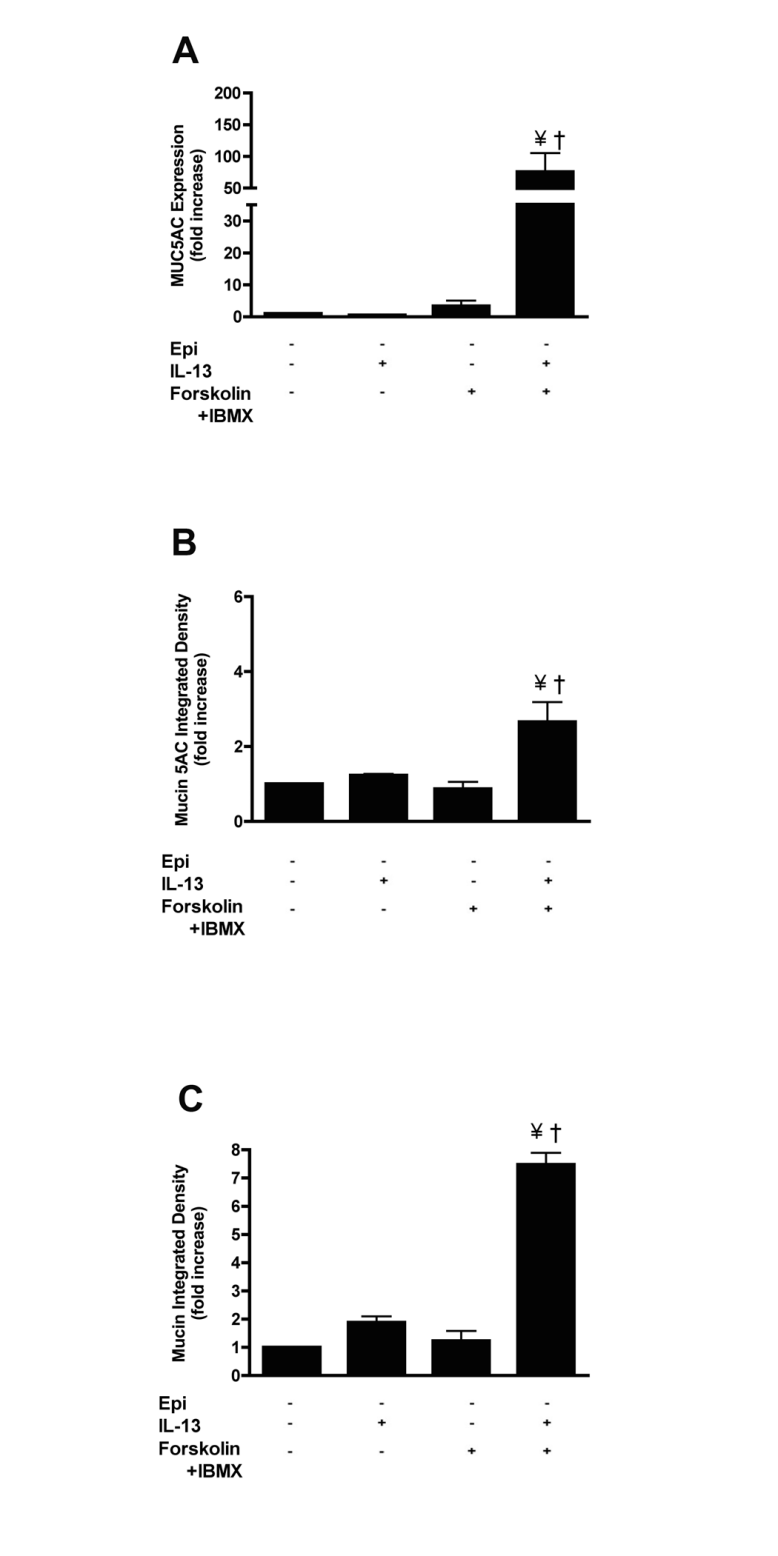
cAMP potentiates mucin production in response to IL-13 in NHBE cells. Cells were grown in the absence of epinephrine, then at ALI, they were incubated with or without 20 ng/ml IL-13 with or without 10 μM forskolin and 100 μM IBMX for 14 days. **A**: MUC5AC transcripts were measured by qRT-PCR, and the data presented as fold change compared to cells grown in the absence of IL-13, IBMX and forskolin (control cells) **B**: Quantification of intracellular mucin 5AC content. The ratio of mucin 5AC integrated density of each group to the integrated density of the cells grown in the presence of epinephrine alone (control cells) was calculated and expressed as fold change. See the supplement for the representative images (S7A Fig). **C**: Quantification of intracellular mucin glycoproteins. The ratio of mucin integrated density and nucleic acid/cytoplasm integrated density was calculated and the data presented as fold change compared to control cells. See the supplement for the representative images (S7B Fig). Data are presented as means ± SEM from three donors. † and ¥ indicate p<0.05 significance as compared to−epinephrine and −epinephrine + IL-13 treated cells respectively.

To provide more evidence for a role for cAMP in mucin production in response to IL-13, we treated cells with 10 μM forskolin combined with 100 μM 3-isobutyl-l-methylxan-thine (IBMX), in the absence of epinephrine. This treatment caused a dramatic increase in MUC5AC expression (75.73 ± 66.59 fold *vs* 0.56 ± 0.40 fold increase by IL-13, p<0.05) (Fig 6A) when the cells were treated with IL-13. The same trend was also observed at the level of intracellular mucin 5AC protein accumulation and mucin content of NHBE cells (Fig 6B and 6C; for representative images see S7A and S7B Fig).

## Discussion

IL-13 plays an important role in the mucus over-production characteristic of bronchial asthma, and MUC5AC is the major mucin gene that is overexpressed by airway epithelium in asthmatic patients [22, 23]. We present evidence that signaling through β_2_AR increases IL-13 induced mucin production in human bronchial epithelium cultured in low RA concentrations. Most previous studies using these cells included epinephrine in the medium (e.g., [10, 18, 24, 25]) and to our knowledge, this is the first study showing an important role for β_2_AR signaling in the IL-13 mediated induction of MUC5AC expression and mucin content in NHBE cells. These results are consistent with our earlier findings that β_2_AR signaling is required for mucous metaplasia in the airways of mice [16, 17, 26]. The clinical relevance of these findings is shown by the ability of formoterol, a commonly used long-acting β-agonist bronchodilator, to mimic epinephrine in the stimulation of MUC5AC transcripts by IL-13 (S2 Fig).

β_2_AR is the principal subtype present in human airway epithelium [27]. To verify that this subtype mediates the effects of IL-13 in cultured NHBE cells, we used preferential βAR antagonists. The selectivity of ICI-118,551 toward β_2_AR, and of CGP 20712A toward β_1_AR is least 500 fold or greater [28]. Treating NHBE cells with the preferential β_2_AR antagonist completely abolished MUC5AC expression in response to IL-13 and epinephrine. In contrast, the preferential β_1_AR antagonist did not alter MUC5AC expression levels under the same conditions. Intracellular mucin 5AC protein levels and mucin glycoprotein were not induced by IL-13 + epinephrine treatment in the presence of ICI-118,551 while CGP 20712A had no effect, paralleling the MUC5AC mRNA expression results (Fig 2A–2C). Our *in vitro* results are also consistent with our recent animal data where genetic ablation of β_2_ARs in mice resulted in attenuation of mucous metaplasia in an allergic murine model of asthma [16, 26].

β_2_AR signaling can proceed from agonist-activated receptors and from constitutively active receptors [29]. Cells that were grown in the absence of epinephrine showed no response to IL-13 treatment in terms of MUC5AC expression, mucin 5AC protein levels and mucin content while cells grown in the presence of epinephrine showed an increase in all three parameters (Fig 1A–1C), suggesting that agonist-induced β_2_AR activation is necessary. To further distinguish between constitutive versus agonist induced β_2_AR signaling, we treated NHBE cells with nadolol or alprenolol in combination with IL-13 in the presence of epinephrine. Nadolol is a non-selective βAR ligand that has full inverse agonist activity at β_2_ARs, thus it blocks both induced and constitutive β_2_AR signaling. Alprenolol lacks inverse agonist activity [29] but has weak β_2_AR agonist activity [30] and behaves as an antagonist in the presence of epinephrine, thus preserving constitutive β_2_AR signaling. Both nadolol and alprenolol blocked the effect of IL-13 on MUC5AC mRNA expression, protein levels and intracellular mucin content to similar extents (Fig 3A–3C). Thus, we provide evidence that constitutive β_2_AR receptor activity is not sufficient to drive the increase in mucin in response to IL-13 in human bronchial epithelial cells. Again, our *in vitro* results are also consistent with our recent mouse data where genetic or pharmacological depletion of epinephrine in mice resulted in attenuation of mucous metaplasia in an antigen-driven murine model of asthma [16, 17, 26]

We have begun determining the basis for the interaction between the β_2_AR and IL-13 signaling pathways. Since β_2_AR can activate MAPKs through cAMP-dependent and independent pathways, we tested the role of these kinases in the expression of MUC5AC. Since cells that were grown in the absence of epinephrine did not show induction of MUC5AC expression by IL-13, we examined only cells grown in the presence of epinephrine. All three families of MAPKs, JNKs, ERKs and p38, are known to be involved in the production of cytokine and chemokines by airway epithelium [31]. Phosphorylated p38 and ERK1/2 are detectable in the epithelium of asthmatic patients and the level of phosphorylation correlates with asthma severity [32]. In NHBE cells, IL-13 induced the phosphorylation of ERK1/2 and the ERK1/2 selective inhibitor, FR180204, attenuated MUC5AC expression, mucin 5AC protein levels and mucin content in response to IL-13 (Fig 4A, 4D–4F). A selective MEK 1/2 inhibitor, U0126, also reduced MUC5AC expression in NHBE cells in response to IL-13 (Nguyen et al, in preparation). These results are consistent with other similar studies in NHBE cells [18].

JNK is involved in regulating pro-inflammatory gene expression and remodeling in airway diseases [33]. In NHBE cells, IL-13 induced the phosphorylation of c-Jun, a distinct JNK downstream target, and a JNK inhibitor, SP600125, inhibited MUC5AC expression and mucin content response to IL-13 (Fig 4B, 4D–4F). Consistent with this finding, attenuation of the asthma phenotype, including mucous metaplasia, by SP600125 has been shown in allergen driven murine model of asthma [34].

The other MAPK that we examined, p38, has four known isoforms: α, β, γ and δ [31]. The transcript levels of the α and β isoforms in the human lung is higher than that of other p38 isoforms of [35]. Inhaled p38α antisense oligonucleotide attenuates mucus production in IL-13 trangenic mice [36]. We did not observe an increase in p38 phosphorylation in response to IL-13 (Fig 4C), however a selective inhibitor of both α and β isoforms [37], SB203580, reduced levels of MUC5AC expression and mucin 5AC content induced by IL-13 and epinephrine (Fig 4D–4F). These data provide evidence for the involvement of p38 in IL-13 induced mucous metaplasia, consisent with previous studies [18, 38]. Even though p38 phosphorylation was not affected by IL-13, basal levels of p38 activation may still be required for MUC5AC expression and mucin 5AC production, perhaps by integrating signals from the β_2_- and IL-13 receptors. There was no significant decrease in total mucin content after inhibiting p38, perhaps because mucin 5AC production decreases while the production of other mucin glycoproteins increases. All of these MAPKs (ERK1/2, JNK and p38) can be activated by β_2_AR *via* the G_s_-adenylyl cyclase pathway, and by G protein-independent, β-arrestin2-dependent pathways [39]. MAPKs then act through downstream components that lead to the activation or translocation to the nucleus of transcriptional factors that act upon the MUC5AC and other genes [40].

To asses the possible involvement of cAMP mediated mechanisms, we used the Rp-isomer of adenosine-3′, 5′-cyclic monophosphorothioate (Rp-cAMPS). Rp-cAMPS binds to PKA preventing its activation by cAMP [41]. MUC5AC expression, intracellular mucin 5AC protein levels and mucin content induced by IL-13 and epinephrine were not significantly inhibited by 50 μM Rp-cAMPS (IC_50_ ~10μM for inhibiting PKA *in vitro*), but were inhibited by 100 μM Rp-cAMPS (Fig 5A–5C). We then examined the effects of raising cAMP levels by treating NHBE cells with forskolin, a direct activator of adenylate cyclase, combined with the non-specific phosphodiesterase inhibitor (PDE), IBMX. This treatment resulted in significant increases in MUC5AC transcripts in response to IL-13 (Fig 6). Taken together the data support a role of cAMP in regulating MUC5AC transcription as induced by IL-13. However, this data must be taken in the context of the high concentration of Rp-cAMPS required for the inhibition of MUC5AC expression, and that we used a compound (forskolin) that causes a ‘global’ intracellular increase in the activity of all adenylate cyclases, and an inhibitor of all PDE subtypes (IBMX) that regulate the breakdown of cAMP. Given the emergence of of data demonstrating distinct subcellular compartmentalization of cAMP associated with specific PDE subtypes [42] we cannot conclusively assert that epinephrine-induced cAMP increases cause the induction of MUC5AC. There are also studies suggesting that, at least with prolonged agonist exposures, other G_s_-coupled receptors such as the adenosine A2B receptor can enhance lung injury and damage epithelial cell integrity [43, 44]. While our experiments could have eliminated a role for cAMP mediating mucin production, the data instead only suggest a role, and additional experiments using more refined pharmacologic tools that are PDE subtype specific, and genetic approaches will be needed to further investigate the possible role of β_2_AR-mediated cAMP increases in regulating mucin production. Given that MAP-kinases can be activated by cAMP mediated mechanisms, it would be interesting to test whether the effect of forskolin + IBMX is negated by inhibition of a MAP kinase, such as ERK or JNK, each of which show increased phosphorylated upon treatment with IL-13 + epinephrine. It may also be suggested that cAMP is needed for p38 to integrate signals from the β_2_- and IL-13 receptors.

How do β_2_AR and IL-13 signaling interact? Due to the multiplicity of pathways proceeding from each receptor, it is not possible at present to firmly identify nodes of cross-talk. However, one potential factor could be an isoform of phosphoinositide 3-kinase (PI3K). Mice deficient in PI3Kγ show reductions in most indices of airway inflammation in OVA-sensitized and challenged mice, though there was disagreement over effects on mucin production [45, 46]. Similar results were obtained, including decreases in mucin production, after treatment of mouse airways with inhibitors of PI3Kγ or δ [47–50]. Studies in NHBE cells showed that pharmacologic inhibition of PI3Kγ reduces IL-13 induced increases in goblet cell density [18]. Possibly, β_2_AR could amplify the IL-13 induction of PI3K isoforms by way of G_i_ [51] or Gβ/γ[52]. An argument also could also be made for p38, which by being constitutive in activity in this system, might therefore be a key transducer in more than one signaling pathway. These possibilities are currently under examination.

In the experiments reported here, only IL-13 and β_2_AR ligands were manipulated, and this reductionist approach does not take into account numerous other cytokines, chemokines and hormones present in the allergic airways. Also, leucocytes and other lung parenchymal cell types are absent from NHBE cultures. Nevertheless, the requirement for β_2_AR signaling in the induction of MUC5AC by IL-13 shown here is consistent with results we have obtained previously with β_2_AR knockout mice, mice chronically treated with β blockers, and mice deficient in epinephrine [16, 17, 26]. A further limitation regards possible off target effects from the use of chemical inhibitors and activators. Future studies utilizing more specific genetic approaches will be needed to delineate the precise signaling pathways involved. In particular, a role for β-arrestin2 cannot be excluded from the present data, and information from *in vivo* studies suggests that there is an involvement of β-arrestin2 in the pathogenesis of asthma [53, 54].

In conclusion, our results reveal an important role for β_2_AR signaling in mediating mucus production in response to IL-13 in NHBE cells. Constitutive β_2_AR activity alone is not sufficient to mediate this effect and it requires agonist activation of the receptor. Moreover, the three major MAPK signaling molecules (ERK1/2, JNK and p38) play a role in mediating the effects of IL-13 in NHBE cells in the presence of epinephrine. Our data also suggests that a cAMP activated signaling cascade may be involved in mediating the inflammatory effect of IL-13. The present report supports the notion that use of selective β_2_AR antagonists could be of value in the treatment of mucus overproduction in asthma and other similar disorders such as COPD [55].

## Supporting Information

S1 Fig Epinephrine is required for mucin production in response to IL-13 in NHBE cell grown with 50 nM retinoic acid. NHBE cells were grown in the presence or absence of 3 μM epinephrine. At ALI, the cells were treated with 20 ng/ml IL-13 for 14 days, total RNA was harvested and then MUC5AC transcripts were measured by qRT-PCR. Data are presented as fold change compared to the corresponding treatment control (in the absence of IL13). *, indicates p<0.05 significance as compared to + epinephrine,—epinephrine and −epinephrine + IL-13 treated cells respectively. N = 3.
(PNG)

S2 Fig Formoterol also potentiates mucin production in response to IL-13. NHBE cells were cultured as described in S1 Fig, except that 10 nM formoterol was used in place of epinephrine.
(PNG)

S3 Fig β_2_ARs are required for mucin production in response to IL-13 in NHBE cells. Representative images of data quantified in Fig 2. **A**: Intracellular MUC5AC content. **B**: Intracellular mucin glycoproteins.
(PNG)

S4 Fig Agonist induced β_2_AR signaling is required for mucin production in response to IL-13 in NHBE cells. Representative images of data quantified in Fig 3. **A**: Intracellular MUC5AC content. **B**: Intracellular mucin glycoproteins.
(PNG)

S5 Fig MAPK signaling is required for mucin production in response to IL-13 in NHBE cells. Representative images of data quantified in Fig 4. **A**: Intracellular MUC5AC content. **B**: Intracellular mucin glycoproteins.
(PNG)

S6 Fig Inhibiting PKA signaling reduced mucin production in response to IL-13 in NHBE cells. Representative images of data quantified in Fig 5. **A**: Intracellular MUC5AC content. **B**: Intracellular mucin glycoproteins.
(PNG)

S7 Fig cAMP potentiates mucin production in response to IL-13 in NHBE cells. Representative images of data quantified in Fig 6. **A**: Intracellular MUC5AC content. **B**: Intracellular mucin glycoproteins.
(PNG)
