# Supplementary figures and images for: Epinephrine Activation of the β2-Adrenoceptor Is Required for IL-13-Induced Mucin Production in Human Bronchial Epithelial Cells
Source: PLoS One. 2015 Jul 10;10(7):e0132559. doi: 10.1371/journal.pone.0132559 (PMC4498766; doi:10.1371/journal.pone.0132559)

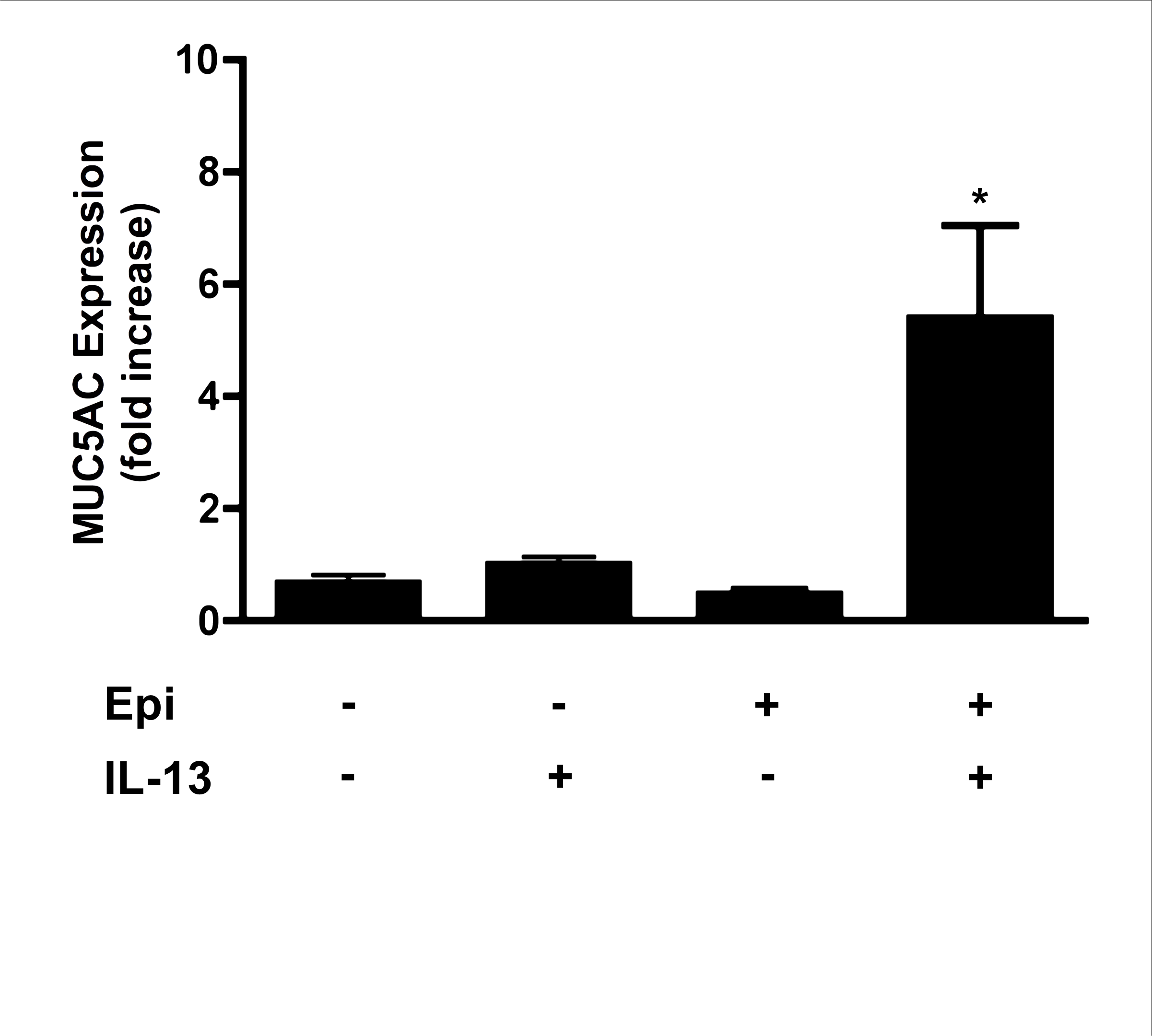

Supplement: S1 Fig — NHBE cells were grown in the presence or absence of 3 μM epinephrine. At ALI, the cells were treated with 20 ng/ml IL-13 for 14 days, total RNA was harvested and then MUC5AC transcripts were measured by qRT-PCR. Data are presented as fold change compared to the corresponding treatment control (in the absence of IL13). *, indicates p<0.05 significance as compared to + epinephrine,—epinephrine and −epinephrine + IL-13 treated cells respectively. N = 3. (PNG) [file pone.0132559.s001.png]

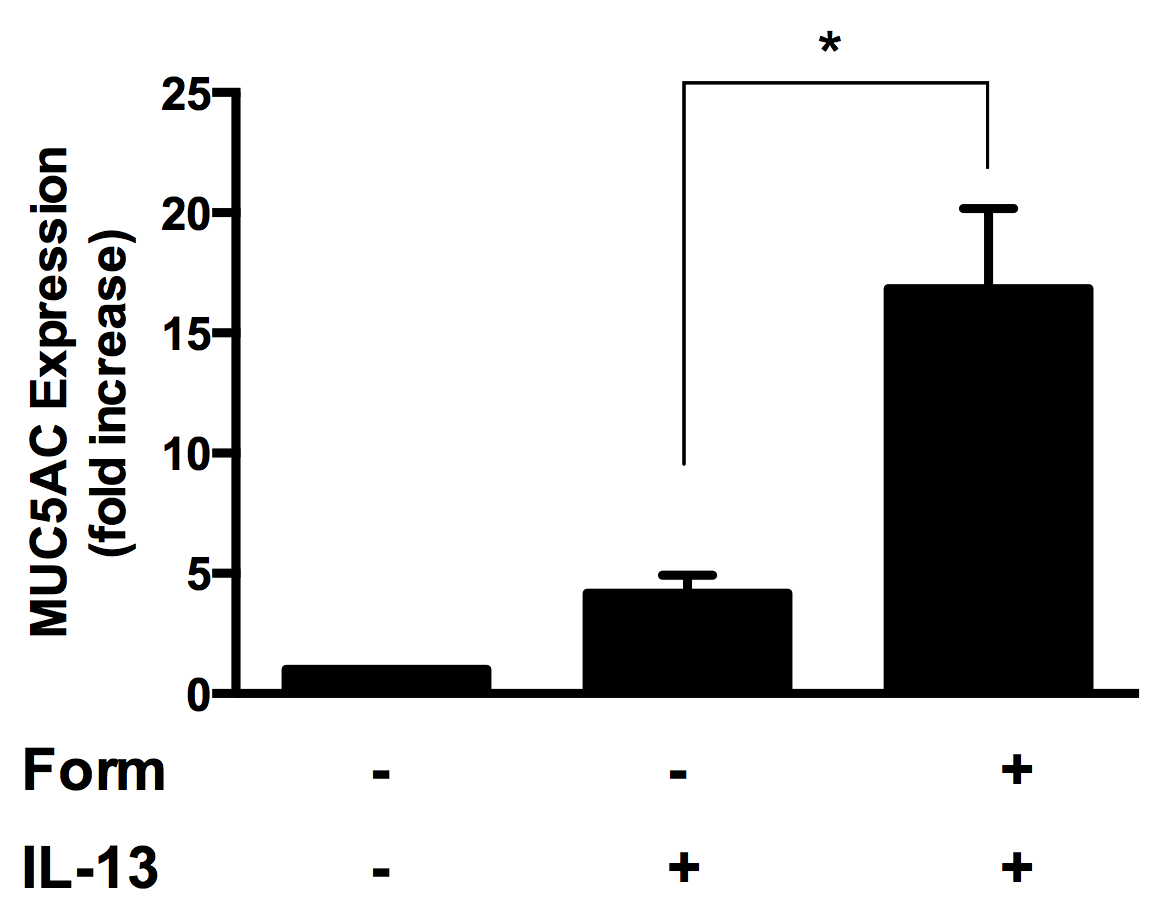

Supplement: S2 Fig — NHBE cells were cultured as described in S1 Fig, except that 10 nM formoterol was used in place of epinephrine. (PNG) [file pone.0132559.s002.png]

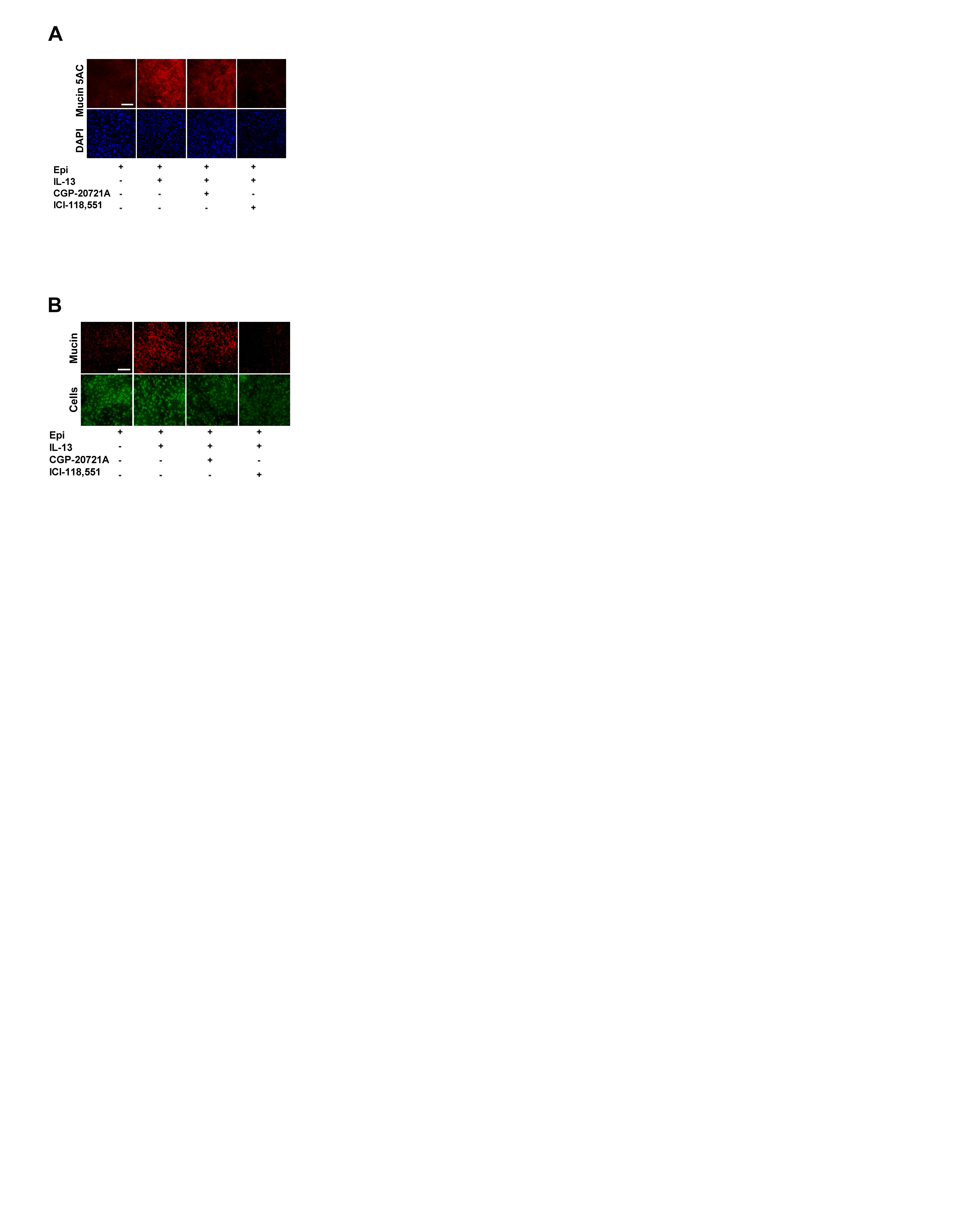

Supplement: S3 Fig — Representative images of data quantified in Fig 2. A: Intracellular MUC5AC content. B: Intracellular mucin glycoproteins. (PNG) [file pone.0132559.s003.png]

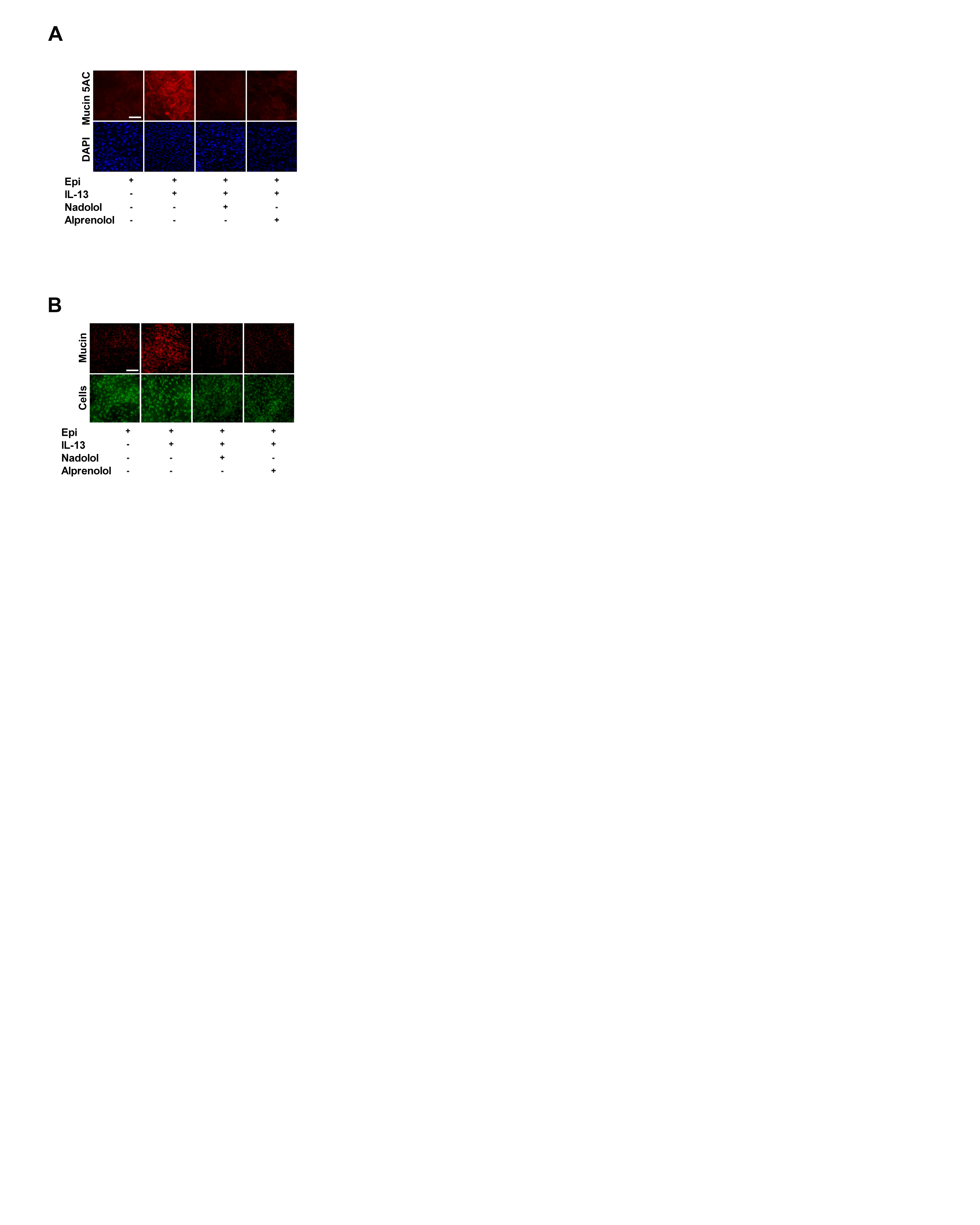

Supplement: S4 Fig — Representative images of data quantified in Fig 3. A: Intracellular MUC5AC content. B: Intracellular mucin glycoproteins. (PNG) [file pone.0132559.s004.png]

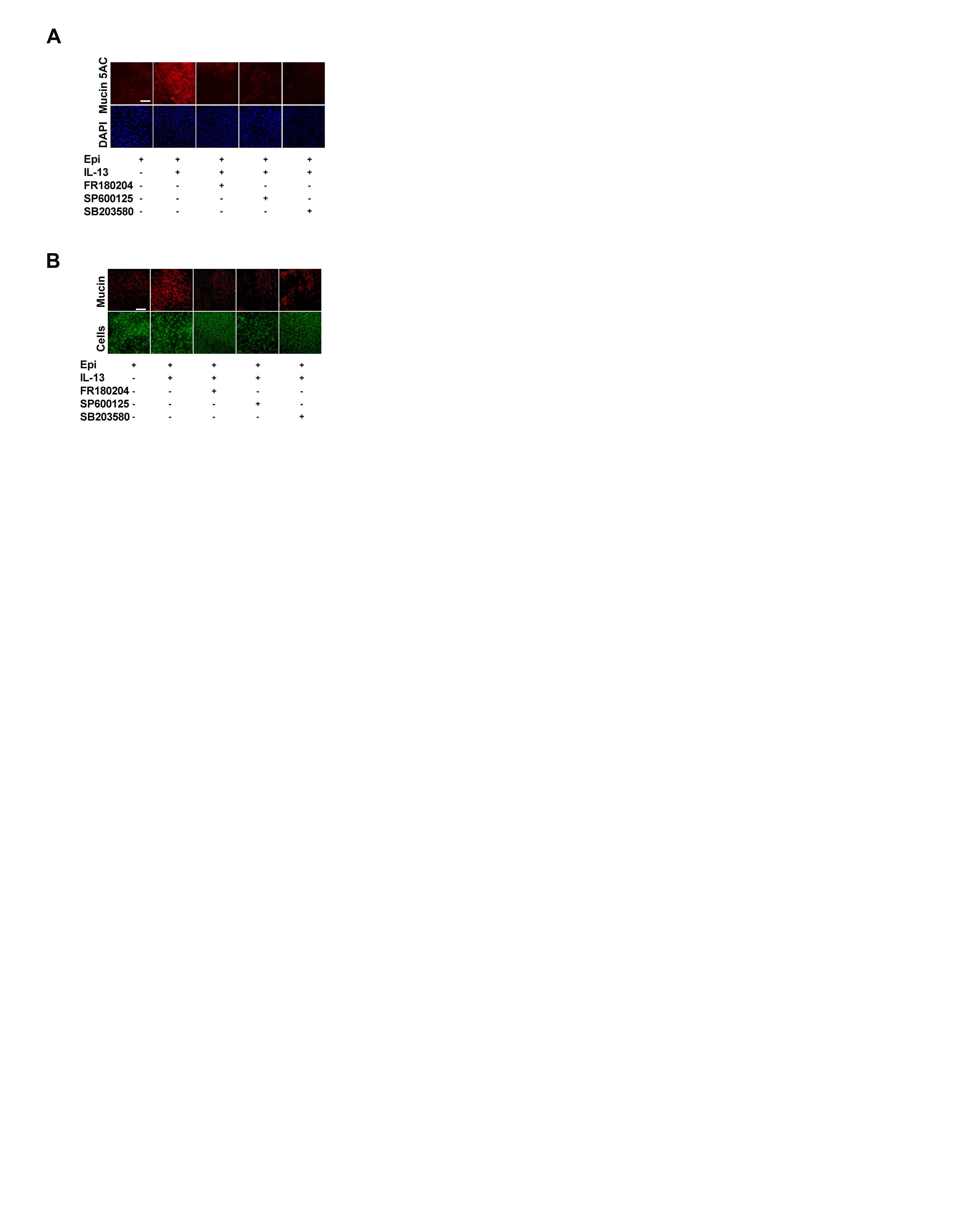

Supplement: S5 Fig — Representative images of data quantified in Fig 4. A: Intracellular MUC5AC content. B: Intracellular mucin glycoproteins. (PNG) [file pone.0132559.s005.png]

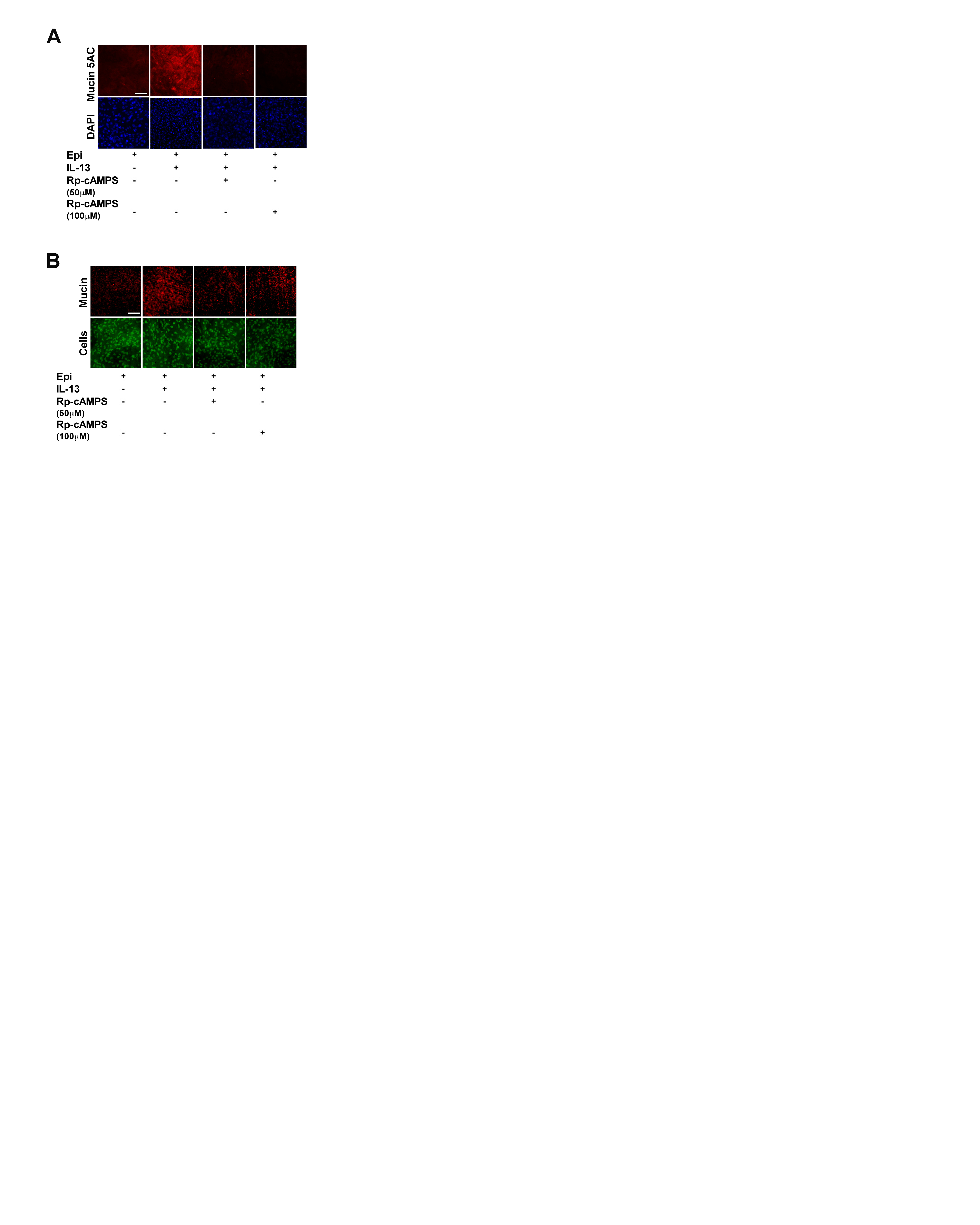

Supplement: S6 Fig — Representative images of data quantified in Fig 5. A: Intracellular MUC5AC content. B: Intracellular mucin glycoproteins. (PNG) [file pone.0132559.s006.png]

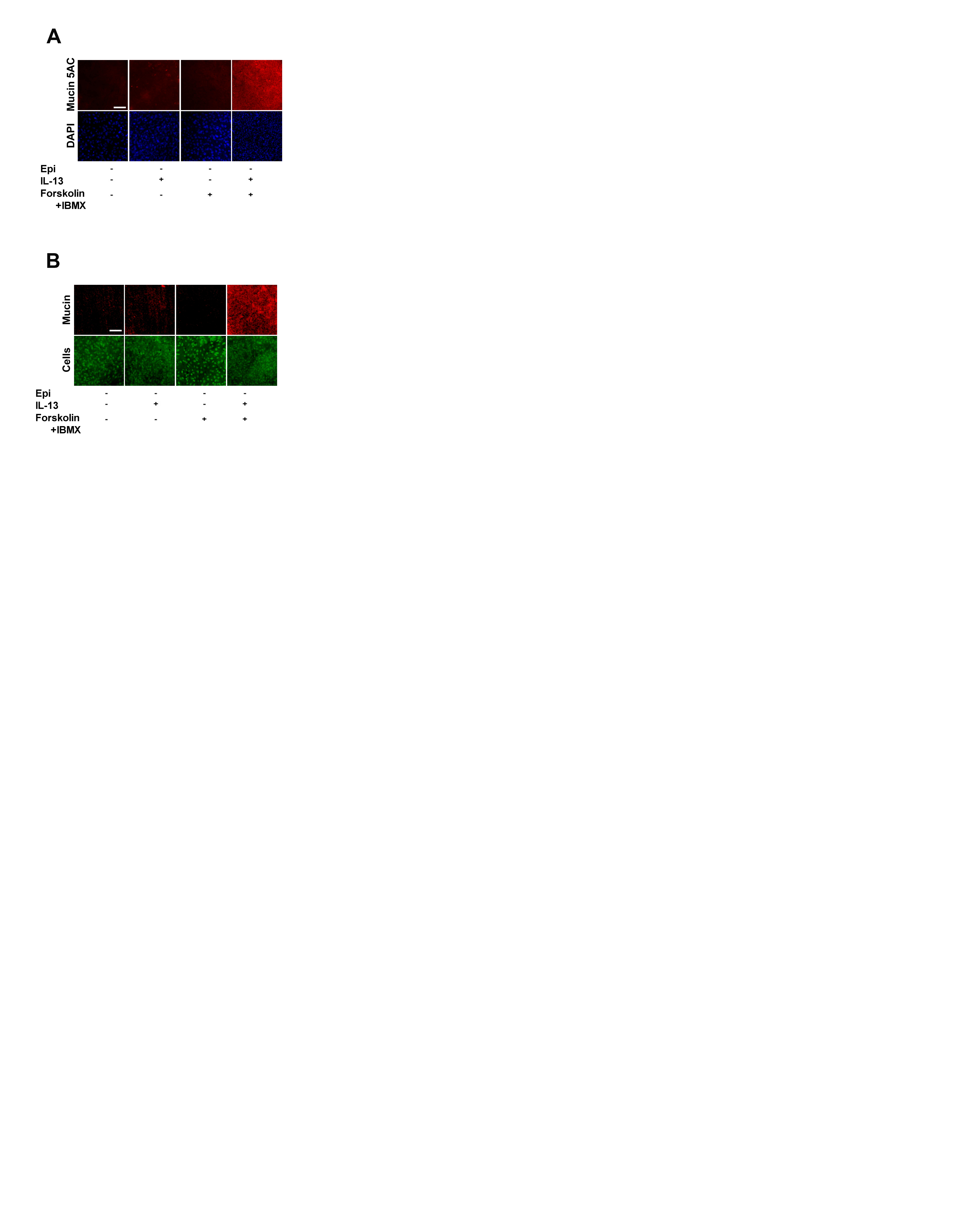

Supplement: S7 Fig — Representative images of data quantified in Fig 6. A: Intracellular MUC5AC content. B: Intracellular mucin glycoproteins. (PNG) [file pone.0132559.s007.png]
